# Selecting Substitutes for Cranial Dural Repair and Preventing Intracranial Iatrogenic Amyloid Transmission

**DOI:** 10.7759/cureus.86487

**Published:** 2025-06-21

**Authors:** Bruno Splavski, Senta Frol

**Affiliations:** 1 Department of Neurosurgery, Dubrovnik General Hospital, Dubrovnik, HRV; 2 Department of Anatomy, Faculty of Applied Health Sciences, University of Zagreb, Zagreb, HRV; 3 Department of Vascular Neurology, University Medical Center, Ljubljana, SVN; 4 Department of Neurology, Faculty of Medicine, University of Ljubljana, Ljubljana, SVN

**Keywords:** cerebral amyloid angiopathy, cranial dural defect, dural substitutes, iatrogenic amyloid transmission, prevention of complications

## Abstract

Dural repair following an osteoplastic craniotomy presents significant challenges, necessitating an optimal dural substitute to avoid complications and ensure successful patient outcomes when primary dural closure is not possible. Ideal substitutes provide a scaffold for fibroblast migration and implantation, which are essential for achieving postoperative dural sealing and watertightness. However, such a substitute seldom exists, underscoring the importance of choosing appropriate materials for dural repair to reduce the risks of postoperative complications, including iatrogenic prion contamination that may promote intracranial amyloid transmission. Available dural substitutes are categorized into autologous, allogeneic, and xenogeneic transplants, as well as organic, synthetic, and composite polymer grafts. While each category offers specific benefits, many disadvantages persist, including the risks of prion-like amyloid protein deposition, particularly spreading iatrogenic Creutzfeldt-Jakob disease. These risks have historically been associated with the use of cadaveric dura grafts and prion-contaminated surgical instruments, necessitating safer replacement materials and enhanced sterilization protocols. This narrative review addresses the critical challenges of dural repair following cranial surgery, proposing innovative directions that include the use of composite materials and emerging technologies, such as 3D printing. Through a narrative review, the evaluation of traditional and advanced dural substitutes is provided, summarizing the advantages and limitations of currently available dural substitutes. In conclusion, the ideal dural substitute should closely replicate the natural structure of the dura, support tissue regeneration, and prevent postoperative complications such as cerebrospinal fluid leakage and intracranial iatrogenic amyloid transmission, thereby ensuring optimal patient outcomes and recovery.

## Introduction and background

Dural repair following cranial surgery is a complex and challenging procedure, making the selection of the optimal substitute for dural repair after an osteoplastic craniotomy critical to ensuring successful patient outcome and recovery when primary dural closure is not possible. The dura mater acts as a natural barrier to intracranial structures. It primarily comprises collagen and elastic fibers arranged into endosteal (outer) and meningeal (inner) layers [1]. Its structural integrity is essential for protecting brain tissue and supporting cerebral neuro-electrical functions.

Cranial dural defects can result from various causes, including traumatic brain injury, extra-axial tumor invasion, dural rupture, and damage induced by cranial surgery or other medical procedures. These defects may lead to postoperative complications such as cerebrospinal fluid (CSF) leakage, intracranial infections (e.g., meningitis), seizures, cerebral herniation, and pseudomeningocele formation [2]. While minor defects can often be repaired spontaneously or with sutures, extensive dural damage necessitates substitutes to expand and restore the dura [3], making a watertight seal to prevent further complications and safeguard the underlying brain tissue.

Dural substitutes provide a framework for fibroblast migration and implantation, which are critical for dura regeneration and synchronized with graft degradation [3]. A perfect dural replacement must restore the dura's integrity and function while exhibiting biological and mechanical properties comparable to or superior to autologous dura transplants [4]. Such substitutes should have biomechanical similarity to human cranial dura. They should exhibit biocompatibility and promote wound healing to prevent CSF leak and minimize cortical scarring by avoiding adhesion formation to surrounding tissues, which may generate seizures [5,6]. They should also be safe to avoid potential intracranial transmission of amyloids and other pathogenic proteins.

Herein, we underscore the importance of selecting appropriate dural substitutes to minimize postoperative complications and ensure favorable surgical outcomes.

## Review

Through a narrative review of the relevant literature, this evaluation provides an overview of traditional and advanced substitutes for cranial dural replacement, summarizing the advantages and limitations of currently available dural substitutes. This evaluation highlights the importance of strengthening the evidence for particular materials used in cranial dural defect repair. The discussion also includes up-to-date information relevant to clinical neurological and neurosurgical practice. The article presents the debate on neurosurgical strategies for dural repair, their historical development, and future perspectives.

Over the last hundred years, various researchers have attempted to repair the dura using a wide range of materials for dural substitutes, primarily derived from animal sources. In 1926, Walter Dandy repaired the dura with autologous fascia lata for the first time [7]. Subsequently, numerous materials for dural substitutes have been documented in the literature, yet no single material has been definitively established as superior. Therefore, choosing an optimal dural substitute remains uncertain [3].

Substitutes for cranial dural defect repair can be categorized into traditional, polymeric, and composite grafts, each offering distinct properties and features. Table 1 summarizes currently available dural substitutes.

**Table 1:** Properties of selected dural substitutes

Type of dural substitute	Advantages	Limitations
Traditional (organic) biomaterials		
Autologous grafts (fascia lata, pericranium)	Non-immunogenic, non-toxic, non-transmissible	Restricted supply. Ineffective in large defects, fragile and delicate, potentially infectious
Allogeneic grafts (human cadaveric dura, amniotic membranes, acellular dermis, small intestine submucosa)	Easily accessible, faster healing, anti-inflammatory	Immunogenic risk, iatrogenic transmission, enhances cortical scarring
Xenogeneic grafts (porcine, bovine, equine pericardium, fish collagen)	Good biocompatibility, low immunogenic risk, stimulates dural regeneration	Potential disease transmission, increased incidence of complications
Non-biological materials		
Natural biopolymers (collagen, silk fibroin, chitosan, bacterial cellulose)	Good biocompatibility, low antigenicity, low host inflammatory response, supports rapid healing	Lack of precise control over degradation, risk of disease transmission, risk of adverse reaction to foreign material
Synthetic polymers, non-degradable (polyurethane), degradable polymers (polycaprolactone, polyglycolic acid, poly L-lactic acid)	Easily accessible, bioinert, non-immunogenic, non-toxic, biocompatible, non-transmissible, customizable	Frail cellular affinity, slow natural healing, reduced cell migration, slow degradation rate, reduced watertightness
Composite integrative allografts (natural + synthetic polymers)	Biodegradable, non-immunogenic, non-toxic, anti-inflammatory, biocompatible, non-transmissible, better cell compatibility, inward tissue growth, neovascularization	Not identified

Traditional grafts are made of biomaterials, including autologous, allogeneic, and xenogeneic materials. Autologous grafts, such as fascia lata and pericranium, are widely utilized due to their non-toxic and non-immunogenic characteristics. However, these grafts are often limited by their unavailability for repairing extensive dural defects [4]. When the fascia lata is concerned, additional surgery is needed, which increases surgical time and donor-site morbidity [8]. The pericranium is a more advantageous option as it eliminates the need for additional donor-site surgery and is readily harvested during craniotomy, reducing the time and cost of surgery [8-10]. Nevertheless, its fragility, delicacy, and short supply make it challenging to handle during surgery [8].

Allogeneic grafts are derived from human tissues, including amniotic membranes, acellular dermis, small intestine submucosa, and cadaveric freeze-dried dura. These materials are widely accessible due to their anti-inflammatory properties, which promote faster healing and reduce the presence of pathogens [10].

However, cadaveric dura allografts have been associated with iatrogenic amyloid transmission risks, particularly prion-like protein deposition linked to iatrogenic Creutzfeldt-Jakob disease (iCJD) [11,12]. Therefore, the transmission of amyloid seeds from cadaveric dural grafting and/or prion-contaminated neurosurgical instruments was suggested as a potential source of disease in some patients [13,14], making the risks associated with prion-like amyloid and other potentially pathogenic protein deposition in the cerebral vessels a substantial concern.

Given the growing evidence of protein transmission mechanisms in neurodegenerative diseases, it remains unclear whether similar propagation involving other pathogenic proteins, such as α-synuclein, along with prions and amyloid, occurs with dura substitutes. However, experimental evidence supports the idea that these proteins gain pathogenicity through a prion-like mechanism, assemble within the nervous system, are influenced by the interaction between the proteopathic agent and the host environment, and may potentially spread beyond the central nervous system [15].

This necessitates a shift to alternative materials, improved sterilization protocols, and the use of single-use surgical instruments to reduce these risks [14]. Consequently, the use of cadaveric grafts has been significantly restricted over the past three decades. Nowadays, alternative allogeneic substitutes, including the amniotic membrane [16], have become more prevalent. Nonetheless, allogeneic grafts may still pose immunogenic risks and enhance cortical scarring.

Xenogeneic substitutes, derived from non-human sources such as porcine, bovine, or equine pericardium and fish collagen, exhibit good biocompatibility and stimulate connective and epithelial tissue to dural regeneration [8]. However, they also carry risks of infection and other postoperative complications, as well as potential disease transmission [3,8].

Non-biological materials, comprising both natural and synthetic polymers, serve as alternatives to traditional grafts. Natural biopolymers, such as collagen, silk fibroin, chitosan, and bacterial cellulose, support rapid healing and exhibit good biocompatibility and low antigenicity, as well as low host inflammatory response [8], but may lack precise control over degradation and carry risks of disease transmission, as well as risks of adverse reaction to foreign material. Synthetic polymers, including non-degradable polyurethane (PUR) and degradable polycaprolactone (PCL), polyglycolic acid (PGA), and poly L-lactic acid (PLLA), are bioinert, non-toxic, and customizable [17,18]. However, they often exhibit weak cell affinity, slower natural healing, reduced cell migration, and lower watertightness [2].

Revising actual case series and systematic reviews on complications related to dural closure using different dura substitutes, numerous clinically relevant moderate-rate complications were observed, including persistent cerebrospinal leakage, aseptic meningitis, seizures, pseudomeningocele formation [2], and hydrodynamic disturbances, together with delayed hydrocephalus. However, those complications were more related to the specific site of the dural defect (like the posterior fossa) than the material used [19]. According to the systematic review provided by Azzam et al. [20], the cumulative complication rate for dural substitutes of all types was 11%.

Recent advancements in composite materials have combined natural and synthetic polymers to address the limitations of other substitutes and further reduce the rate of post-procedural complications. Composites such as collagen/PLGA/chitosan and PLA/PCL/collagen exhibit controlled biodegradability and desirable physicochemical, mechanical, and biological properties, making them promising candidates for dural substitutes [4,21]. These materials are also non-immunogenic, non-toxic, and non-transmissible, exhibiting better cell compatibility, inward tissue growth, and neovascularization, and almost meeting the criteria of an ideal dural replacement [22].

At the end of this paper, it is necessary to address the limitations that arise from its narrative review character. Therefore, continued research through larger multicentric studies is essential for a better understanding of the clinical and pathological characteristics of available and future substitutes for cranial dural repair.

## Conclusions

Cranial dural substitutes commonly employed in neurosurgical clinical practice include autologous, allogeneic, and xenogeneic transplants, as well as organic and synthetic polymeric grafts. An ideal dural substitute replicates the natural structure of the dura, supports tissue regeneration, and effectively prevents CSF leakage, as well as other postoperative complications, including the risk of infection. It should also address safety concerns to reduce the transmission of intracranial iatrogenic pathogenic proteins.

Clinicians must remain vigilant about the possibility of intracranial iatrogenic amyloid transmission, which highlights the need for proactive measures to reduce its incidence. Revisiting dural graft selection standards and prioritizing safer, biocompatible materials should be an urgent priority in the neurosurgical community. To overcome the shortcomings of existing substitutes, composite materials that integrate organic and synthetic polymers, combined with advancements in 3D printing and regenerative medicine, show significant promise for improving surgical outcomes and the safety of cranial dural repair.
